# Carotid sinus massage for diagnosis in narrow QRS tachycardia

**DOI:** 10.1007/s12471-015-0731-4

**Published:** 2015-07-17

**Authors:** I.R. Henkens, K. Zeppenfeld, A.D. Hauer

**Affiliations:** 1 Department of Cardiology, Bravis Hospital, Location Roosendaal, The Netherlands; 2 Department of Heart Diseases, Leiden University Medical Center, Leiden, The Netherlands; 3 Department of Cardiology, HAGA Hospital, The Hague, The Netherlands

A 50-year-old woman without a prior medical history was referred to the emergency room by a general practitioner because of palpitations. Two hours before, the patient had experienced her heart suddenly beating faster, but was otherwise feeling well. She was not using any medicine and denied substance abuse. Physical examination did not reveal any abnormalities except for a high pulse frequency. The ECG in Fig. 1 was recorded while performing carotid sinus massage.

**Fig. 1:**
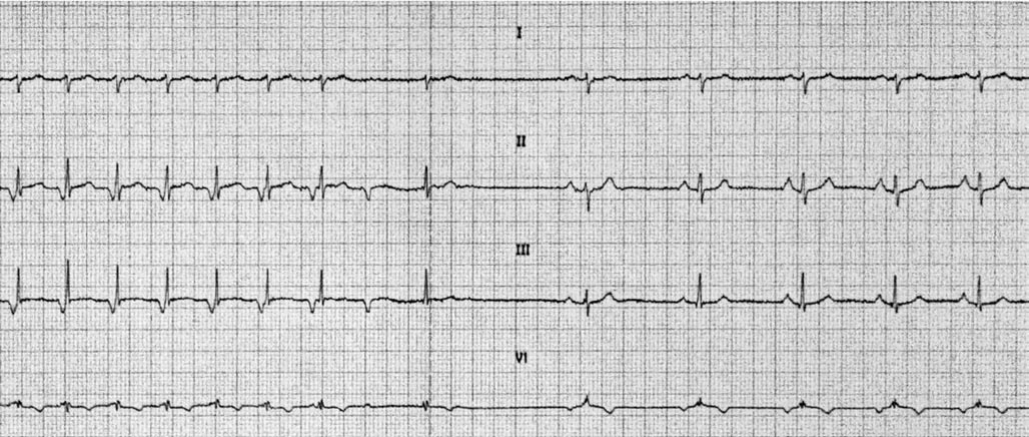
ECG during carotid sinus massage

## Questions

1. What is the most likely diagnosis of the tachycardia?

2. What is the likely origin of the eighth QRS complex?

3. What is the potential explanation for the morphology of the ninth QRS complex?

## Answer

You will find the answer elsewhere in this issue.

